# Single or in Combination Antimicrobial Resistance Mechanisms of *Klebsiella pneumoniae* Contribute to Varied Susceptibility to Different Carbapenems

**DOI:** 10.1371/journal.pone.0079640

**Published:** 2013-11-12

**Authors:** Yu-Kuo Tsai, Ci-Hong Liou, Chang-Phone Fung, Jung-Chung Lin, L. Kristopher Siu

**Affiliations:** 1 National Institute of Infectious Diseases and Vaccinology, National Health Research Institutes, Miaoli, Taiwan; 2 Division of Infectious Diseases and Tropical Medicine, Department of Internal Medicine, Tri-Service General Hospital, National Defense Medical Center, Taipei, Taiwan; 3 Section of Infectious Diseases, Department of Medicine, Taipei Veterans General Hospital and National Yang-Ming University, Taipei, Taiwan; 4 Graduate Institute of Basic Medical Science, China Medical University, Taichung, Taiwan; Quuen's University Belfast, United Kingdom

## Abstract

Resistance to carbapenems has been documented by the production of carbapenemase or the loss of porins combined with extended-spectrum β-lactamases or AmpC β-lactamases. However, no complete comparisons have been made regarding the contributions of each resistance mechanism towards carbapenem resistance. In this study, we genetically engineered mutants of *Klebsiella pneumoniae* with individual and combined resistance mechanisms, and then compared each resistance mechanism in response to ertapenem, imipenem, meropenem, doripenem and other antibiotics. Among the four studied carbapenems, ertapenem was the least active against the loss of porins, cephalosporinases and carbapenemases. In addition to the production of KPC-2 or NDM-1 alone, resistance to all four carbapenems could also be conferred by the loss of two major porins, OmpK35 and OmpK36, combined with CTX-M-15 or DHA-1 with its regulator AmpR. Because the loss of OmpK35/36 alone or the loss of a single porin combined with *bla*_CTX-M-15_ or *bla*_DHA-1_-*ampR* expression was only sufficient for ertapenem resistance, our results suggest that carbapenems other than ertapenem should still be effective against these strains and laboratory testing for non-susceptibility to other carbapenems should improve the accurate identification of these isolates.

## Introduction

The increasing prevalence of extended-spectrum β-lactamases (ESBLs) and plasmid-mediated AmpC β-lactamases in *Enterobacteriaceae* is a critical concern for scientists trying to develop treatments against bacterial infections. TEM-, SHV- and CTX-M-type ESBLs and CMY- and DHA-type AmpC β-lactamases are commonly found in multidrug-resistant *Enterobacteriaceae* [1-4]. Carbapenem has been recommended as an effective drug for these strains. However, carbapenem-resistant *Enterobacteriaceae* have recently been reported worldwide. In *Klebsiella pneumoniae*, numerous reports have confirmed carbapenem resistance by ESBLs or AmpC β-lactamases combined with the loss of outer membrane porins OmpK35 and/or OmpK36 [5-7], or by carbapenemases alone [8].

Currently, four carbapenems are used clinically (imipenem, meropenem, ertapenem and doripenem). The mechanisms of carbapenem resistance were mainly detected from clinical isolates with individual or combined genetic alterations. No comparisons have been made on all four carbapenems and on the contributions of each resistance mechanism towards carbapenem resistance. In the present study, we created mutants with individual or combined resistance mechanisms from a susceptible clinical *K. pneumoniae* isolate and studied each resistance mechanism in response to these four carbapenems.

## Materials and Methods

### Bacterial strains, plasmids and growth conditions

The bacterial strains and plasmids used in this study are listed in Table 1. The *K. pneumoniae* clinical strains were isolated from different patients in Taiwan or America, and strain NVT2001 has been found to be resistant only to ampicillin [9]. The low-copy-number plasmid pACYC177 was used in the cloning experiments. *Escherichia coli* and *K. pneumoniae* strains were cultured at 37°C in Mueller-Hinton broth (MHB) or Luria-Bertani (LB) broth with the appropriate antibiotics. 

**Table 1:** Bacterial strains and plasmids used in this study.

**Strain or plasmid**	**Description^a^**	**Source or reference**
Strains		
*K. pneumoniae*		
ZAT242	Clinical isolate with plasmid pCT	This study
CGMHKL1	Clinical isolate with plasmid pSH	This study
VGC263	Clinical isolate with plasmid pDH	This study
KPC2010	Clinical isolate with plasmid pKP	[11]
TAI2010	Clinical isolate with plasmid pND	[27]
NVT2001S	Streptomycin-resistant isolate of clinical strain NVT2001	[9]
Δ*ompK35* mutant	*ompK35* deletion strain of NVT2001S; Sm^r^	[9]
Δ*ompK36* mutant	*ompK36* deletion strain of NVT2001S; Sm^r^	[9]
Δ*ompK35/36* mutant	*ompK35* and *ompK36* deletion strain of NVT2001S; Sm^r^	[9]
*E. coli*		
J53	A recipient for conjugation experiment; Az^r^	[28]
Plasmids		
pACYC177	Low-copy-number plasmid; Ap^r^ Km^r^	New England Biolabs
pCT	Plasmid from clinical isolate containing *bla*_CTX-M-15_	This study
pSH	Plasmid from clinical isolate containing *bla*_SHV-12_	This study
pDH	Plasmid from clinical isolate containing *bla*_DHA-1_-*ampR*	This study
pKP	Plasmid from clinical isolate containing *bla*_KPC-2_ and *bla*_TEM-1_	[11]
pND	Plasmid from clinical isolate containing *bla*_NDM-1_	[27]
pCT177	Fragment containing *bla*_CTX-M-15_ and its flanking region cloned into pACYC177	This study
pSH177	Fragment containing *bla*_SHV-12_ and its flanking region cloned into pACYC177	This study
pDH177	Fragment containing *bla*_DHA-1_-*ampR* and its flanking region cloned into pACYC177	This study
pDHA177	Fragment containing *bla*_DHA-1_ and its flanking region cloned into pACYC177	This study
pAmpR177	Fragment containing *ampR* and its flanking region cloned into pACYC177	This study
pKP177	Fragment containing *bla*_KPC-2_ and its flanking region cloned into pACYC177	This study
pND177	Fragment containing *bla*_NDM-1_ and its flanking region cloned into pACYC177	This study

^a^Ap^r^, resistance to ampicillin; Az^r^, resistance to azide; Km^r^, resistance to kanamycin; Sm^r^, resistance to streptomycin.

### Conjugation experiments and plasmid content

Direct transfer of the β-lactamase-carrying plasmids from *K. pneumoniae* clinical isolates to azide-resistant *E. coli* J53 was performed by filter conjugation [10]. Transconjugants were selected on LB agar plates containing sodium azide (100 μg/ml) for counterselection and cefotaxime (1 μg/ml) for selection of plasmid-encoded resistance. Because *E. coli* J53 does not retain the *bla*_KPC-2_-carrying plasmids of clinical *K. pneumoniae* for any period of time [11], the *bla*_KPC-2_-carrying plasmid was directly transferred to the *K. pneumoniae* strains NVT2001S, Δ*ompK35*, Δ*ompK36* and Δ*ompK35/36* by filter conjugation [10]. Transconjugants were selected on LB agar plates containing streptomycin (500 μg/ml) for counterselection and cefotaxime (0.5–2 μg/ml) for selection of plasmid-encoded resistance. Resistance genes, including *bla*_CTX-M_, *bla*_SHV_, *bla*_TEM_, *bla*_DHA_, *bla*_CMY_, *bla*_MOX_, *bla*_LAT_, *bla*_BIL_, *bla*_KPC_ and *bla*_NDM_, were detected among the transconjugant plasmids using PCR with specific primers (Table S1) and then further confirmed by sequencing. All the sequence analyses were conducted using the NCBI database (http://www.ncbi.nlm.nih.gov/). Isolates carrying resistance genes that had previously been sequence-confirmed were used as positive controls in every PCR assay.

### Cloning and strain construction

DNA fragments of the *bla*_CTX-M-15_, *bla*_SHV-12_, *bla*_DHA-1_-*ampR*, *bla*_DHA-1_, *ampR*, *bla*_KPC-2_ and *bla*_NDM-1_ genes with their flanking regions were amplified from transconjugants by PCR with specific primers (Table S1). The generated PCR fragments were digested with *Pst*I or *Bam*HI and then cloned into pACYC177. The resulting plasmids were then transformed into the *K. pneumoniae* strains NVT2001S, Δ*ompK35*, Δ*ompK36* and Δ*ompK35/36* by electroporation. The recombinant bacteria were plated onto LB agar plates containing kanamycin (25 μg/ml), and the presence of the β-lactamase genes was confirmed by PCR and sequencing. Direct transfer of the β-lactamase-carrying plasmids from *E. coli* J53 transconjugants to the *K. pneumoniae* strains NVT2001S, Δ*ompK35*, Δ*ompK36* and Δ*ompK35/36* was performed by filter conjugation [10]. Transconjugants were selected on brilliant green containing inositol-nitrate-deoxycholate (BIND) plates containing cefotaxime (1 μg/ml) for the selection of plasmid-encoded resistance, and the growth of non-*K. pneumoniae* strains was effectively suppressed on BIND plates [12]. The presence of the β-lactamase genes was verified by PCR and sequencing.

### Antimicrobial susceptibility test

Minimal inhibitory concentrations (MICs) of ertapenem, imipenem, meropenem and doripenem were determined using the E-test (Biodisk AB, Sweden). MICs of the other 15 antimicrobial agents were determined using a broth microdilution test according to the recommendations of the clinical and laboratory standards institute (CLSI) [13]. The following antimicrobial agents were used: aztreonam, ampicillin, piperacillin/tazobactam, cefazolin, cephalothin, cefoxitin, ceftriaxone, cefpodoxime, cefotaxime, cefotaxime/clavulanic acid, ceftazidime, ceftazidime/clavulanic acid, cefepime, ciprofloxacin and gentamicin. All the results were interpreted according to the breakpoints established by the CLSI in 2013 [14].

## Results

### Strain construction and porin loss in antibiotic resistance

The β-lactamases detected in the clinical plasmids are shown in Table 1, while *bla*_CTX-M-15_, *bla*_SHV-12_, *bla*_DHA-1_-*ampR*, *bla*_KPC-2_ or *bla*_NDM-1_ was found in each of the five clinical plasmids. To evaluate the effect of these β-lactamases alone and in combination with porin loss on antimicrobial resistance, the clinical and recombinant plasmids were transferred into the NVT2001S strain and its porin-loss mutants. The loss of OmpK35 alone did not significantly influence the antibiotic resistance of the NVT2001S strain (Table 2). Conversely, the loss of OmpK36 alone conferred resistance to cefazolin, cephalothin and cefoxitin, and the loss of OmpK35/36 caused highly resistance against these cephalosporins (Table 2).

**Table 2:** MICs of antibiotics against *K. pneumoniae* NVT2001S and its porin-loss mutants with or without the low-copy-number plasmid pACYC177.

**Antibiotic^a^**	**Minimal inhibitory concentration (μg/ml)^b^**							
	**NVT2001S**		**Δ*ompK35***		**Δ*ompK36***		**Δ*ompK35/36***	
	**NO^c^**	**LCP**	**NO**	**LCP**	**NO**	**LCP**	**NO**	**LCP**
Aztreonam	≤1	≤1	≤1	≤1	≤1	≤1	≤1	≤1
Ampicillin	≥32	≥32	≥32	≥32	≥32	≥32	≥32	≥32
Piperacillin/TZB	≤4	≤4	≤4	≤4	≤4	≤4	≤4	≤4
Cefazolin	≤8	≤8	≤8	≤8	16	16	≥**32**	≥**32**
Cephalothin	≤8	≤8	≤8	≤8	16	16	≥**32**	≥**32**
Cefoxitin	≤4	≤4	≤4	≤4	**16**	**16**	**32**	**32**
Ceftriaxone	≤1	≤1	≤1	≤1	≤1	≤1	≤1	≤1
Cefpodoxime	≤0.25	≤0.25	≤0.25	≤0.25	≤0.25	≤0.25	0.5	0.5
Cefotaxime	≤0.25	≤0.25	≤0.25	≤0.25	≤0.25	≤0.25	≤0.25	≤0.25
Cefotaxime/CLA	≤0.12	≤0.12	≤0.12	≤0.12	≤0.12	≤0.12	0.25	0.25
Ceftazidime	≤0.25	≤0.25	≤0.25	≤0.25	≤0.25	≤0.25	0.5	0.5
Ceftazidime/CLA	≤0.12	≤0.12	0.25	0.25	≤0.12	≤0.12	**0.5**	**0.5**
Cefepime	≤1	≤1	≤1	≤1	≤1	≤1	≤1	≤1
Ciprofloxacin	≤1	≤1	≤1	≤1	≤1	≤1	≤1	≤1
Gentamicin	≤4	≤4	≤4	≤4	≤4	≤4	≤4	≤4

^a^TZB, tazobactam with a fixed concentration of 4 μg/ml; CLA, clavulanic acid with a fixed concentration of 4 μg/ml.

^b^Boldface numbers indicate a significant (≥4-fold) difference in the MICs of *K. pneumoniae* NVT2001S and its derived strains, while the underlined numbers were above the breakpoint of susceptibility established by CLSI in 2013 [14].

^c^No, no supplemental plasmid; LCP, low-copy-number plasmid.

### ESBLs with or without porin loss in antibiotic resistance

The production of CTX-M-15 or SHV-12 alone conferred resistance to aztreonam and many of the cephalosporins tested (Table 3). With the loss of OmpK36, the CTX-M-15 and SHV-12 strains showed resistance to aztreonam, piperacillin/tazobactam and all the cephalosporins tested, while some β-lactam/β-lactamase inhibitor combinations were still effective against these strains (Table 3).

**Table 3:** MICs of antibiotics against *K. pneumoniae* NVT2001S and its porin-loss mutants with extended-spectrum β-lactamase.

**Antibiotic^a^**	**Minimal inhibitory concentration (μg/ml)^b^**															
	**NVT2001S**				**Δ*ompK35***				**Δ*ompK36***				**Δ*ompK35/36***			
	**CTX-M-15^c^**		**SHV-12**		**CTX-M-15**		**SHV-12**		**CTX-M-15**		**SHV-12**		**CTX-M-15**		**SHV-12**	
	**pCT^d^**	**LCP**	**pSH**	**LCP**	**pCT**	**LCP**	**pSH**	**LCP**	**pCT**	**LCP**	**pSH**	**LCP**	**pCT**	**LCP**	**pSH**	**LCP**
Aztreonam	**64**	**128**	≥**256**	**128**	≥**256**	≥**256**	≥**256**	≥**256**	**128**	≥**256**	≥**256**	≥**256**	≥**256**	≥**256**	≥**256**	≥**256**
Ampicillin	≥32	≥32	≥32	≥32	≥32	≥32	≥32	≥32	≥32	≥32	≥32	≥32	≥32	≥32	≥32	≥32
Piperacillin/TZB	**16**	≤4	≥**128**	8	**16**	**16**	≥**128**	≥**128**	≥**128**	≥**128**	≥**128**	≥**128**	≥**128**	≥**128**	≥**128**	≥**128**
Cefazolin	≥**32**	≥**32**	≥**32**	≥**32**	≥**32**	≥**32**	≥**32**	≥**32**	≥**32**	≥**32**	≥**32**	≥**32**	≥**32**	≥**32**	≥**32**	≥**32**
Cephalothin	≥**32**	≥**32**	≥**32**	≥**32**	≥**32**	≥**32**	≥**32**	≥**32**	≥**32**	≥**32**	≥**32**	≥**32**	≥**32**	≥**32**	≥**32**	≥**32**
Cefoxitin	≤4	≤4	≤4	≤4	≤4	≤4	≤4	≤4	**16**	**32**	**32**	**16**	**64**	**32**	**32**	**32**
Ceftriaxone	≥**256**	≥**256**	**128**	**16**	≥**256**	≥**256**	**128**	**32**	≥**256**	≥**256**	≥**256**	**64**	≥**256**	≥**256**	≥**256**	≥**256**
Cefpodoxime	≥**64**	≥**64**	≥**64**	**32**	≥**64**	≥**64**	≥**64**	**32**	≥**64**	≥**64**	≥**64**	≥**64**	≥**64**	≥**64**	≥**64**	≥**64**
Cefotaxime	≥**128**	≥**128**	**32**	**16**	≥**128**	≥**128**	**32**	**16**	≥**128**	≥**128**	≥**128**	**64**	≥**128**	≥**128**	≥**128**	≥**128**
Cefotaxime/CLA	≤0.12	≤0.12	≤0.12	≤0.12	≤0.12	≤0.12	≤0.12	≤0.12	0.25	**0.5**	≤0.12	≤0.12	**2**	**4**	**0.5**	**0.5**
Ceftazidime	**16**	**64**	≥**256**	**128**	**32**	**128**	≥**256**	≥**256**	**32**	**128**	≥**256**	≥**256**	**128**	≥**256**	≥**256**	≥**256**
Ceftazidime/CLA	0.25	**0.5**	**0.5**	0.25	0.25	**1**	**1**	**0.5**	0.25	**1**	**2**	**0.5**	**1**	**4**	**8**	**8**
Cefepime	**16**	≥**32**	**8**	2	≥**32**	≥**32**	**8**	2	≥**32**	≥**32**	≥**32**	**16**	≥**32**	≥**32**	≥**32**	≥**32**
Ciprofloxacin	≤1	≤1	≤1	≤1	≤1	≤1	≤1	≤1	≤1	≤1	≤1	≤1	≤1	≤1	≤1	≤1
Gentamicin	≥**32**	≤4	≤4	≤4	≥**32**	≤4	≤4	≤4	≥**32**	≤4	≤4	≤4	≥**32**	≤4	≤4	≤4

^a^TZB, tazobactam with a fixed concentration of 4 μg/ml; CLA, clavulanic acid with a fixed concentration of 4 μg/ml.

^b^Boldface numbers indicate a significant (≥4-fold) difference in the MICs of *K. pneumoniae* NVT2001S and its derived strains, while the underlined numbers were above the breakpoint of susceptibility established by CLSI in 2013 [14].

^c^The β-lactamase on the plasmid was shown, and the plasmid was transferred into *K. pneumoniae* NVT2001S and its porin-loss mutants.

^d^pCT and pSH, plasmids from clinical isolates; LCP, low-copy-number plasmid.

### AmpC β-lactamases with or without porin loss in antibiotic resistance

NVT2001S harboring the clinical plasmid with *bla*_DHA-1_-*ampR* showed significantly (≥4-fold) higher MICs for several antibiotics tested compared to that harboring the recombinant plasmid with *bla*_DHA-1_-*ampR*. This result indicates that other resistant genes should exist on the clinical plasmid (Table 4). For the strains harboring the recombinant plasmid, the expression of *bla*_DHA-1_-*ampR* could confer significantly (≥4-fold) higher MICs of many antibiotics tested compared to the expression of *bla*_DHA-1_ alone. No significant (≥4-fold) differences in MICs were found between the AmpR strain and its parental strain (Tables 4). With the loss of OmpK35/36, the DHA-1-AmpR strains were highly resistant to aztreonam, piperacillin/tazobactam and all the cephalosporins tested except cefepime (Table 4).

**Table 4:** MICs of antibiotics against *K. pneumoniae* NVT2001S and its porin-loss mutants with AmpC β-lactamase and/or its regulator.

**Antibiotic^a^**	**Minimal inhibitory concentration (μg/ml)^b^**															
	**NVT2001S**				**Δ*ompK35***				**Δ*ompK36***				**Δ*ompK35/36***			
	**DHA-1-AmpR^c^**		**DHA-1**	**AmpR**	**DHA-1-AmpR**		**DHA-1**	**AmpR**	**DHA-1-AmpR**		**DHA-1**	**AmpR**	**DHA-1-AmpR**		**DHA-1**	**AmpR**
	**pDH^d^**	**LCP**	**LCP**	**LCP**	**pDH**	**LCP**	**LCP**	**LCP**	**pDH**	**LCP**	**LCP**	**LCP**	**pDH**	**LCP**	**LCP**	**LCP**
Aztreonam	**16**	2	≤1	≤1	**64**	**16**	**4**	≤1	**32**	**4**	≤1	≤1	≥**256**	**64**	**4**	≤1
Ampicillin	≥32	≥32	≥32	≥32	≥32	≥32	≥32	≥32	≥32	≥32	≥32	≥32	≥32	≥32	≥32	≥32
Piperacillin/TZB	≥**128**	8	≤4	≤4	≥**128**	8	≤4	≤4	≥**128**	8	≤4	≤4	≥**128**	≥**128**	8	≤4
Cefazolin	≥**32**	≥**32**	16	≤8	≥**32**	≥**32**	≥**32**	≤8	≥**32**	≥**32**	≥**32**	≤8	≥**32**	≥**32**	≥**32**	≥**32**
Cephalothin	≥**32**	≥**32**	≥**32**	≤8	≥**32**	≥**32**	≥**32**	≤8	≥**32**	≥**32**	≥**32**	16	≥**32**	≥**32**	≥**32**	≥**32**
Cefoxitin	≥**128**	≥**128**	≤4	≤4	≥**128**	≥**128**	8	≤4	≥**128**	≥**128**	**32**	**16**	≥**128**	≥**128**	**64**	**32**
Ceftriaxone	**8**	2	≤1	≤1	**8**	**4**	≤1	≤1	**64**	**8**	2	≤1	**128**	**16**	**4**	≤1
Cefpodoxime	≥**64**	≥**64**	**8**	≤0.25	≥**64**	**32**	**8**	≤0.25	≥**64**	≥**64**	**32**	0.5	≥**64**	≥**64**	≥**64**	0.5
Cefotaxime	**32**	**8**	**2**	≤0.25	**32**	**8**	**2**	≤0.25	≥**128**	**32**	**8**	≤0.25	≥**128**	**64**	**16**	≤0.25
Cefotaxime/CLA	**64**	**32**	**1**	≤0.12	**64**	**32**	**1**	≤0.12	≥**128**	≥**128**	**8**	0.25	≥**128**	≥**128**	**8**	0.25
Ceftazidime	≥**256**	**32**	**8**	≤0.25	≥**256**	**64**	**16**	0.5	≥**256**	**64**	**16**	≤0.25	≥**256**	**128**	**32**	0.5
Ceftazidime/CLA	≥**256**	**128**	**4**	≤0.12	≥**256**	≥**256**	**8**	0.25	≥**256**	**128**	**8**	0.25	≥**256**	≥**256**	**32**	**0.5**
Cefepime	≤1	≤1	≤1	≤1	≤1	≤1	≤1	≤1	2	≤1	≤1	≤1	**4**	≤1	≤1	≤1
Ciprofloxacin	≤1	≤1	≤1	≤1	≤1	≤1	≤1	≤1	≤1	≤1	≤1	≤1	≤1	≤1	≤1	≤1
Gentamicin	**16**	≤4	≤4	≤4	**16**	≤4	≤4	≤4	**16**	≤4	≤4	≤4	≥**32**	≤4	≤4	≤4

^a^TZB, tazobactam with a fixed concentration of 4 μg/ml; CLA, clavulanic acid with a fixed concentration of 4 μg/ml.

^b^Boldface numbers indicate a significant (≥4-fold) difference in the MICs of *K. pneumoniae* NVT2001S and its derived strains, while the underlined numbers were above the breakpoint of susceptibility established by CLSI in 2013 [14].

^c^The β-lactamase and/or its regulator on the plasmid were shown, and the plasmid was transferred into *K. pneumoniae* NVT2001S and its porin-loss mutants.

^d^pDH, plasmid from clinical isolate; LCP, low-copy-number plasmid.

### Carbapenemases with or without porin loss in antibiotic resistance

In all the antibiotics tested, the KPC-2 strains showed susceptibility to ciprofloxacin and gentamicin. The NDM-1 strains showed susceptibility to aztreonam, ciprofloxacin and gentamicin, except gentamicin for the strains harboring the clinical plasmid with *bla*_NDM-1_ (Table 5). The loss of porins did not increase the resistance of these strains to these antibiotics (Table 5).

**Table 5:** MICs of antibiotics against *K. pneumoniae* NVT2001S and its porin-loss mutants with carbapenemase.

**Antibiotic^a^**	**Minimal inhibitory concentration (μg/ml)^b^**															
	**NVT2001S**				**Δ*ompK35***				**Δ*ompK36***				**Δ*ompK35/36***			
	**KPC-2^c^**		**NDM-1**		**KPC-2**		**NDM-1**		**KPC-2**		**NDM-1**		**KPC-2**		**NDM-1**	
	**pKP^d^**	**LCP**	**pND**	**LCP**	**pKP**	**LCP**	**pND**	**LCP**	**pKP**	**LCP**	**pND**	**LCP**	**pKP**	**LCP**	**pND**	**LCP**
Aztreonam	≥**256**	≥**256**	≤1	≤1	≥**256**	≥**256**	≤1	≤1	≥**256**	≥**256**	≤1	≤1	≥**256**	≥**256**	≤1	≤1
Ampicillin	≥32	≥32	≥32	≥32	≥32	≥32	≥32	≥32	≥32	≥32	≥32	≥32	≥32	≥32	≥32	≥32
Piperacillin/TZB	≥**128**	≥**128**	≥**128**	≥**128**	≥**128**	≥**128**	≥**128**	≥**128**	≥**128**	≥**128**	≥**128**	≥**128**	≥**128**	≥**128**	≥**128**	≥**128**
Cefazolin	≥**32**	≥**32**	≥**32**	≥**32**	≥**32**	≥**32**	≥**32**	≥**32**	≥**32**	≥**32**	≥**32**	≥**32**	≥**32**	≥**32**	≥**32**	≥**32**
Cephalothin	≥**32**	≥**32**	≥**32**	≥**32**	≥**32**	≥**32**	≥**32**	≥**32**	≥**32**	≥**32**	≥**32**	≥**32**	≥**32**	≥**32**	≥**32**	≥**32**
Cefoxitin	≤4	**16**	≥**128**	≥**128**	8	**32**	≥**128**	≥**128**	**64**	≥**128**	≥**128**	≥**128**	≥**128**	≥**128**	≥**128**	≥**128**
Ceftriaxone	**16**	**64**	≥**256**	≥**256**	**16**	**64**	≥**256**	≥**256**	**64**	≥**256**	≥**256**	≥**256**	≥**256**	≥**256**	≥**256**	≥**256**
Cefpodoxime	**16**	≥**64**	≥**64**	≥**64**	**16**	≥**64**	≥**64**	≥**64**	≥**64**	≥**64**	≥**64**	≥**64**	≥**64**	≥**64**	≥**64**	≥**64**
Cefotaxime	**2**	**16**	≥**128**	≥**128**	**4**	**16**	≥**128**	≥**128**	**64**	≥**128**	≥**128**	≥**128**	≥**128**	≥**128**	≥**128**	≥**128**
Cefotaxime/CLA	**0.5**	**8**	**64**	≥**128**	**1**	**8**	≥**128**	≥**128**	**16**	**64**	≥**128**	≥**128**	**64**	≥**128**	≥**128**	≥**128**
Ceftazidime	**8**	**32**	≥**256**	≥**256**	**16**	**64**	≥**256**	≥**256**	**32**	**64**	≥**256**	≥**256**	**64**	≥**256**	≥**256**	≥**256**
Ceftazidime/CLA	**2**	**32**	≥**256**	≥**256**	**4**	**64**	≥**256**	≥**256**	**8**	**64**	≥**256**	≥**256**	**64**	≥**256**	≥**256**	≥**256**
Cefepime	2	**16**	**16**	≥**32**	**4**	**16**	**16**	≥**32**	**16**	≥**32**	≥**32**	≥**32**	≥**32**	≥**32**	≥**32**	≥**32**
Ciprofloxacin	≤1	≤1	≤1	≤1	≤1	≤1	≤1	≤1	≤1	≤1	≤1	≤1	≤1	≤1	≤1	≤1
Gentamicin	≤4	≤4	≥**32**	≤4	≤4	≤4	≥**32**	≤4	≤4	≤4	≥**32**	≤4	≤4	≤4	≥**32**	≤4

^a^TZB, tazobactam with a fixed concentration of 4 μg/ml; CLA, clavulanic acid with a fixed concentration of 4 μg/ml.

^b^Boldface numbers indicate a significant (≥4-fold) difference in the MICs of *K. pneumoniae* NVT2001S and its derived strains, while the underlined numbers were above the breakpoint of susceptibility established by CLSI in 2013 [14].

^c^The β-lactamase on the plasmid was shown, and the plasmid was transferred into *K. pneumoniae* NVT2001S and its porin-loss mutants.

^d^pKP and pND, plasmids from clinical isolate; LCP, low-copy-number plasmid.

### Single or in combination antimicrobial resistance mechanisms in carbapenem resistance

The loss of OmpK35 or OmpK36 alone did not significantly (≥4-fold) influence carbapenem resistance of the NVT2001S strain. The loss of OmpK35/36 conferred 31-, 8- and 4-fold increases in the MICs of ertapenem, meropenem and doripenem, respectively, and led to ertapenem resistance (Table 6). Without porin loss, the CTX-M-15 and DHA-1-AmpR strains only showed a significant (≥4-fold) increase in the ertapenem MIC, while the KPC-2 and NDM-1 strains exhibited resistance to all four carbapenems (Table 6). With the loss of OmpK36, the CTX-M-15 and DHA-1-AmpR strains became resistant to ertapenem. With the loss of OmpK35/36, the CTX-M-15, SHV-12 and DHA-1-AmpR strains became resistant to all four carbapenems, except imipenem and doripenem for the strain harboring the recombinant plasmid with *bla*_SHV-12_ (Table 6). 

**Table 6:** MICs of carbapenems against *K. pneumoniae* NVT2001S and its derived strains.

**Strain and carbapenem**	**Minimal inhibitory concentration (μg/ml)^a^**													
	**None**		**CTX-M-15^b^**		**SHV-12**		**DHA-1-AmpR**		**DHA-1**	**AmpR**	**KPC-2**		**NDM-1**	
	**No^c^**	**LCP**	**pCT**	**LCP**	**pSH**	**LCP**	**pDH**	**LCP**	**LCP**	**LCP**	**pKP**	**LCP**	**pND**	**LCP**
NVT2001S														
Ertapenem	0.032	0.047	**0.19**	**0.38**	**0.125**	0.047	**0.75**	**0.38**	0.047	0.012	**12**	>**32**	**24**	>**32**
Imipenem	0.25	0.25	0.25	0.25	0.38	0.38	0.25	0.25	0.25	0.25	**8**	>**32**	**16**	>**32**
Meropenem	0.047	0.047	0.064	0.125	0.064	0.047	0.064	0.064	0.047	0.032	**8**	>**32**	**12**	>**32**
Doripenem	0.047	0.047	0.064	0.094	0.047	0.047	0.064	0.064	0.047	0.047	**3**	>**32**	**12**	>**32**
Δ*ompK35*														
Ertapenem	0.032	0.047	**0.5**	**0.5**	**0.25**	0.094	**1.0**	**0.75**	**0.125**	0.023	**16**	>**32**	**32**	>**32**
Imipenem	0.25	0.25	0.25	0.25	0.38	0.38	0.25	0.25	0.25	0.25	**12**	>**32**	**24**	>**32**
Meropenem	0.064	0.047	0.094	0.125	0.094	0.064	0.094	0.094	0.064	0.047	**8**	>**32**	**24**	>**32**
Doripenem	0.047	0.047	0.064	0.125	0.064	0.047	0.094	0.094	0.047	0.047	**4**	>**32**	**16**	>**32**
Δ*ompK36*														
Ertapenem	0.047	0.047	**1.0**	**1.0**	**0.38**	**0.125**	**4**	**2**	**0.19**	0.023	>**32**	>**32**	>**32**	>**32**
Imipenem	0.25	0.25	0.38	0.5	0.38	0.38	**1.0**	0.5	0.5	0.25	>**32**	>**32**	>**32**	>**32**
Meropenem	0.064	0.064	**0.19**	**0.25**	**0.19**	0.094	**0.5**	**0.38**	0.064	0.047	>**32**	>**32**	>**32**	>**32**
Doripenem	0.047	0.047	0.125	**0.25**	0.125	0.094	**0.5**	**0.38**	0.125	0.047	>**32**	>**32**	>**32**	>**32**
Δ*ompK35/36*														
Ertapenem	**1.0**	**1.5**	>**32**	>**32**	>**32**	**12**	>**32**	>**32**	**4**	**1.0**	>**32**	>**32**	>**32**	>**32**
Imipenem	0.5	0.5	**2**	**3**	**2**	0.75	**32**	**12**	**1.0**	0.38	>**32**	>**32**	>**32**	>**32**
Meropenem	**0.38**	**0.5**	**6**	**8**	**4**	**1.5**	**24**	**8**	**0.75**	**0.38**	>**32**	>**32**	>**32**	>**32**
Doripenem	**0.19**	**0.25**	**3**	**6**	**2**	**1.0**	**16**	**8**	**0.38**	0.125	>**32**	>**32**	>**32**	>**32**

^a^Boldface numbers indicate a significant (≥4-fold) difference in the MICs of *K. pneumoniae* NVT2001S and its derived strains, while the underlined numbers were above the breakpoint of susceptibility established by CLSI in 2013 [14].

^b^The β-lactamase and/or its regulator on the plasmid were shown, and the plasmid was transferred into *K. pneumoniae* NVT2001S and its porin-loss mutants.

^c^No, no supplemental plasmid; pCT, pSH, pDH, pKP and pND, plasmids from clinical isolates; LCP, low-copy-number plasmid.

## Discussion

Of the four carbapenems in this study, the loss of OmpK35/36 alone could confer ertapenem resistance, and the expression of *bla*_CTX-M-15_ or *bla*_DHA-1_-*ampR* alone was only sufficient to significantly (≥4-fold) increase the ertapenem MIC. Previous studies have shown that CTX-M β-lactamase activity against ertapenem is very low. CTX-M likely contributes to the decreased ertapenem susceptibility by binding with a high affinity to this molecule [15,16]. Conversely, the production of KPC-2 or NDM-1 alone could render *K. pneumoniae* strains resistant to all four carbapenems, and the ertapenem MIC was the highest. Our results suggest that among the four carbapenems, ertapenem is the least active against the loss of porins, cephalosporinases and carbapenemases.

Previous studies have found that carbapenem resistance in clinical isolates can be conferred by porin loss combined with the production of ESBLs or AmpC β-lactamases. In particular, ertapenem resistance can be caused by porin loss with the CTX-M variants [17,18]. Our results further revealed that resistance to all four carbapenems could be rendered by the loss of OmpK35/36 combined with the expression of *bla*_CTX-M-15_ or *bla*_DHA-1_-*ampR* in *K. pneumoniae*. Because carbapenems are frequently utilized as drugs of last resort for the treatment of a variety of infections caused by multidrug-resistant bacteria, this finding is notable when using carbapenems for treating infections due to ESBL- or AmpC β-lactamase-producing *Enterobacteriaceae* with the loss of porins. 

DHA-1 is a plasmid-encoded AmpC β-lactamase and *bla*_DHA-1_ expression is transcriptionally regulated by the divergently read *ampR* gene [19]. The mechanism of AmpC induction is intimately linked to a cell wall recycling system [20-22]. AmpR can both activate and repress *ampC* expression according to its interaction with specific murein degradation products, and the murein synthesis was interfered with β-lactams [3,21]. Previous study showed that AmpR represses the synthesis of AmpC β-lactamase by 2.5-fold in the absence of an inducer, while its expression is induced more than 10-fold in the presence of a β-lactam [23]. The β-lactams also differ in their inducing abilities [3]. Of the strains harboring recombinant plasmids in this study, the DHA-1-AmpR strains showed higher MICs for many antibiotics tested compared with those of the DHA-1 strains. Whether this result is due to the expression of *bla*_DHA-1_ induced by AmpR in the presence of these antibiotics requires further studies. The DHA-1-AmpR strains with the loss of OmpK35/36 became highly resistant to multiple drugs, including the four carbapenems. However, cefepime should be an effective β-lactam against these strains.

Carbapenemase can effectively inactivate most β-lactam antibiotics, including carbapenems, and most carbapenemase genes are transferable. The rapid identification of carbapenemase producers is needed to prevent the development of outbreaks. Ertapenem is a sensitive indicator for detecting most of the carbapenemase producers [24,25]. However, ertapenem showed lower specificity for detecting carbapenemases in clinical isolates compared with imipenem and meropenem [26]. Of the four carbapenems in this study, our results also showed that the loss of OmpK35/36 or the loss of a single porin combined with the expression of *bla*_CTX-M-15_ or *bla*_DHA-1_-*ampR* was only sufficient to lead to ertapenem resistance. Because resistance to all four carbapenems could be conferred by the loss of OmpK35/36 combined with the expression of *bla*_CTX-M-15_ or *bla*_DHA-1_-*ampR*, special tests for carbapenemase detection should be applied, especially in regions where these β-lactamase-producing strains are endemic.

## Supporting Information

Table S1 **Oligonucleotide primers used in this study.**
(DOC)
