# Supplementary material for: Single or in Combination Antimicrobial Resistance Mechanisms of Klebsiella pneumoniae Contribute to Varied Susceptibility to Different Carbapenems
Source: PLoS One. 2013 Nov 12;8(11):e79640. doi: 10.1371/journal.pone.0079640 (PMC3827147; doi:10.1371/journal.pone.0079640)
Supplement: Table S1 — Oligonucleotide primers used in this study. (DOC) [file pone.0079640.s001.doc]

**Table S1.** Oligonucleotide primers used in this study.

| Target | Primer | Sequence (5′-3′)*^a^* | Expected amplicon | Reference or GenBank no. |
| --- | --- | --- | --- | --- |
| CTX-M group 1 | CTX-1F | AAAAATCACTGCGCCAGTTC | 415 bp | [1] |
|  | CTX-1R | AGCTTATTCATCGCCACGTT |  | [1] |
| CTX-M group 2 | CTX-2F | CGACGCTACCCCTGCTATT | 552 bp | [1] |
|  | CTX-2R | CCAGCGTCAGATTTTTCAGG |  | [1] |
| CTX-M group 9 | CTX-9F | CAAAGAGAGTGCAACGGATG | 205 bp | [1] |
|  | CTX-9R | ATTGGAAAGCGTTCATCACC |  | [1] |
| CTX-M group 8/25 | CTX-8F | TCGCGTTAAGCGGATGATGC | 666 bp | [1] |
|  | CTX-25F | GCACGATGACATTCGGG | 327 bp | [1] |
|  | CTX-8/25R | AACCCACGATGTGGGTAGC |  | [1] |
| SHV | SHV-F | GGGTTATTCTTATTTGTCGC | 931 bp | [2] |
|  | SHV-R | TTAGCGTTGCCAGTGCTC |  | [2] |
| TEM | TEM-F | ATAAAATTCTTGAAGACGAAA | 1080 bp | [2] |
|  | TEM-R | GACAGTTACCAATGCTTAATCA |  | [2] |
| DHA-1, DHA-2 | DHA-MF | AACTTTCACAGGTGTGCTGGGT | 405 bp | [3] |
|  | DHA-MR | CCGTACGCATACTGGCTTTGC |  | [3] |
| MOX-1, MOX-2, CMY-1,  CMY-8 to CMY-11 | MOX-MF | GCTGCTCAAGGAGCACAGGAT | 520 bp | [3] |
|  | MOX-MR | CACATTGACATAGGTGTGGTGC |  | [3] |
| LAT-1 to LAT-4, CMY-2  to CMY-7, BIL-1 | CIT-MF | TGGCCAGAACTGACAGGCAAA | 462 bp | [3] |
|  | CIT-MR | TTTCTCCTGAACGTGGCTGGC |  | [3] |
| KPC | KPC-F | ATGTCACTGTATCGCCGTCT | 893 bp | [4] |
|  | KPC-R | TTTTCAGAGCCTTACTGCCC |  | [4] |
| NDM-1 | NDM-Fm | GGTTTGGCGATCTGGTTTTC | 621 bp | [5] |
|  | NDM-Rm | CGGAATGGCTCATCACGATC |  | [5] |
| CTX-M-15 and  its flanking region | CTX-pstF | AAACTgCAgTTgAgTgTTgCTCTgTgg (*Pst*I) | 1191 bp | HQ157356 |
|  | CTX-pstR | AAACTgCAgCACTTTTgCCgTCTAA (*Pst*I) |  | HQ157356 |
| SHV-12 and  its flanking region | SHV-bamF | cgggatcctgccgtatttgcagtaccag (*Bam*HI) | 1226 bp | GU553923 |
|  | SHV-bamR | cgggatccacgtttatggcgttacct (*Bam*HI) |  | GU553923 |
| DHA-1–AmpR and  its flanking region | AmpR-pstF | AAACTgCAGcctgccgtgataccgtg (*Pst*I) | 2456 bp | AY705809 |
|  | DHA-pstR | AAACTgCAgCTgTCAgTgCCCgATA (*Pst*I) |  | AY705809 |
| DHA-1 and  its flanking region | DHA-pstF | AAACTgCAgggAgATAACgTCTgACCA (*Pst*I) | 1405 bp | AY705809 |
|  | DHA-pstR | AAACTgCAgCTgTCAgTgCCCgATA (*Pst*I) |  | AY705809 |
| AmpR and  its flanking region | AmpR-pstF | AAACTgCAGcctgccgtgataccgtg (*Pst*I) | 1327 bp | AY705809 |
|  | AmpR-pstR | AAACTgCAGATCCTGCTGTGCCATCA (*Pst*I) |  | AY705809 |
| KPC-2 and  its flanking region | KPC-bamF | cgggatccaagaggaaggcgggagtg (*Bam*HI) | 1481 bp | JX430448 |
|  | KPC-bamR | cgggatccGAGGGCGAAGGTTAAATGG (*Bam*HI) |  | JX430448 |
| NDM-1 and  its flanking region | NDM-pstF | AAACTgCAgACTCgTCCTAgAAAggCgTTAg (*Pst*I) | 1127 bp | HQ451074 |
|  | NDM-pstR | AAACTgCAgTCCAACTCgTCgCAAAgC (*Pst*I) |  | HQ451074 |

*^a^* The restriction sites designed in the oligonucleotide are underlined, with the corresponding endonucleases indicated in parentheses.

**References**

1. Woodford N, Fagan EJ, Ellington MJ (2006) Multiplex PCR for rapid detection of genes encoding CTX-M extended-spectrum β-lactamases. J Antimicrob Chemother 57: 154-155.
2. Chang FY, Siu LK, Fung CP, Huang MH, Ho M (2001) Diversity of SHV and TEM β-lactamases in *Klebsiella pneumoniae*: gene evolution in Northern Taiwan and two novel β-lactamases, SHV-25 and SHV-26. Antimicrob Agents Chemother 45: 2407-2413.
3. Perez-Perez FJ, Hanson ND (2002) Detection of plasmid-mediated AmpC β-lactamase genes in clinical isolates by using multiplex PCR. J Clin Microbiol 40: 2153-2162.
4. Bradford PA, Bratu S, Urban C, Visalli M, Mariano N, et al. (2004) Emergence of carbapenem-resistant *Klebsiella* species possessing the class A carbapenem-hydrolyzing KPC-2 and inhibitor-resistant TEM-30 β-lactamases in New York City. Clin Infect Dis 39: 55-60.
5. Nordmann P, Poirel L, Carrer A, Toleman MA, Walsh TR (2011) How to detect NDM-1 producers. J Clin Microbiol 49: 718-721.
